# Expression of a Grapevine NAC Transcription Factor Gene Is Induced in Response to Powdery Mildew Colonization in Salicylic Acid-Independent Manner

**DOI:** 10.1038/srep30825

**Published:** 2016-08-04

**Authors:** Zsofia Toth, Patrick Winterhagen, Balazs Kalapos, Yingcai Su, Laszlo Kovacs, Erzsebet Kiss

**Affiliations:** 1 Institute of Genetics and Biotechnology, Szent Istvan University, 2100-Godollo, Hungary; 2 Institute of Crop Science, University of Hohenheim, 70599-Stuttgart, Germany; 3 Agricultural Institute, Centre for Agricultural Research, Hungarian Academy of Sciences, 2462-Martonvasar, Hungary; 4 Department of Mathematics, Missouri State University, 65897-Springfield, USA; 5 Department of Biology, Missouri State University, 65897-Springfield, USA

## Abstract

Tissue colonization by grape powdery mildew (PM) pathogen *Erysiphe necator* (Schw.) Burr triggers a major remodeling of the transcriptome in the susceptible grapevine *Vitis vinifera* L. While changes in the expression of many genes bear the signature of salicylic acid (SA) mediated regulation, the breadth of PM-induced changes suggests the involvement of additional regulatory networks. To explore PM-associated gene regulation mediated by other SA-independent systems, we designed a microarray experiment to distinguish between transcriptome changes induced by *E. necator* colonization and those triggered by elevated SA levels. We found that the majority of genes responded to both SA and PM, but certain genes were responsive to PM infection alone. Among them, we identified genes of stilbene synthases, PR-10 proteins, and several transcription factors. The microarray results demonstrated that the regulation of these genes is either independent of SA, or dependent, but SA alone is insufficient to bring about their regulation. We inserted the promoter-reporter fusion of a PM-responsive transcription factor gene into a wild-type and two SA-signaling deficient *Arabidopsis* lines and challenged the resulting transgenic plants with an *Arabidopsis*-adapted PM pathogen. Our results provide experimental evidence that this grape gene promoter is activated by the pathogen in a SA-independent manner.

*E. necator* (Schwein) Burr is a biotrophic ascomycetous fungus which causes PM disease on grapevine and other species of the *Vitaceae* family^1^,^2^. The pathogen colonizes photosynthetically active tissues of susceptible plants by penetrating the cuticle and epidermal cell wall and forming specialized feeding structures, named haustoria, inside the cell lumen. In order to colonize its host, *E. necator* must suppress the first layer of the host defense system. PMs, as other obligate pathogens, accomplish this by secreting effector proteins into the cytoplasm of invaded host cells. Many putative effectors have been recently identified in other PM pathogens. For example, the genome of the PM fungus *Blumeria graminis*, adapted to infect grasses, contains 491 genes for candidates of secreted effector proteins, 43 of which have been detected in plant cells surrounding haustoria^3^. Recently, 8 of these effector proteins have been shown to be *bona fide* effectors^4^.

Plant species that co-evolved with their adapted PM pathogens express nucleotide-binding domain leucine-rich repeat receptors (*NLRs*) which recognize the activity of effector proteins and signal to the host cell nucleus^5^. This effector recognition triggers hypersensitive response (HR) at the site of infection and a substantial up-regulation of defense-related genes in the surrounding and distal tissues. The process that leads to HR is referred to as effector-triggered immunity^6^. Induction of defense-related gene expression in distal tissues is termed systemic acquired resistance and is believed to be mediated by SA, a stress hormone that is a key component of the defense signaling pathway against biotrophic pathogens^7^. Recent evidence that PM resistance in the North American wild grape *Muscadinia rotundifolia* requires a NLR-type gene^8^ suggests that effector-triggered immunity operates through a similar mechanism in grapevine^9^.

Interestingly, PM substantially up-regulates of many defense-related genes in susceptible grapevine also, as demonstrated by our earlier results of global microarray^10^ and SSH studies^11^. These experiments have revealed changes in the transcription of a broad range of genes not typically associated with defense. This suggests that PM infection brings about gene expression changes in the host which regulate processes other than defense. Previous studies in *Arabidopsis thaliana* suggested that many of these genes are unlikely to be directed by SA signaling^12^,^13^, but regulated probably by other signals, such as hydrogen peroxide, ethylene, jasmonic acid, or by fungal elicitors. The magnitude of the transcriptional changes of PM-induced but SA-independent genes has been described by Chandran and co-workers^13^ who performed comparative global transcriptome analysis in the *Golovinomyces-Arabidopsis* pathosystem using wild-type and SA biosynthesis mutant *isc1-2* host plants. They demonstrated that the expression pattern of 62% of the genes responsive to PM was the same in wild-type as in the *isc1-2* plants, indicating that, at most, only 38% of PM-triggered gene-regulation is SA-dependent. Furthermore, of the 47 PM-responsive regulatory genes in the wild-type, 17 were unaffected in expression by the *isc1* mutation, suggesting that a substantial component of the PM-triggered transcriptome remodeling program does not require SA signaling. Other mechanisms may also play a regulatory role during PM infection: for example, activation of many defense-related genes is accompanied by H_2_O_2_ production and a peak in H_2_O_2_ levels has been registered in PM-infected grapevine also^10^,^14^.

The identification of PM-responsive genes in SA-independent regulation has provided novel insights into the molecular mechanism by which PM pathogens establish interaction with their hosts^13^. Several compatibility genes that are required for enhancement of PM infection have been shown to be regulated in this way. A well-known example of a compatibility gene displaying increased expression in PM-infected tissues is the *MLO* gene in barley^15^. As in barley, *MLO* genes were found to be induced by *E. necator* infection in *V. vinifera* in order to assist penetration by the adapted PM fungus^16^,^17^,^18^. Although, the *MLO*s are believed to be stimulated via Ca^2+^/calmodulin-mediated signaling, a subset of *VvMLOs* was found to be SA-inducible, suggesting that SA may feedback regulate the role of *MLOs* in defense^18^. In barley and tomato varieties homozygous recessive genotypes have provided durable and broad-spectrum resistance against PM pathogens^19^, and therefore, a thorough knowledge of PM-responsive genes and the understanding of their regulation may potentially lead to engineering other forms of durable resistance in crop plants.

Our aim was to identify grapevine genes that are responsive to advanced PM infection independent of SA. Using microarray analysis, we examined the grapevine transcriptome in leaves with mature *E. necator* colonies and in leaves with elevated SA levels induced by methyl salicylate (MeSA) treatment. By overlaying the two resulting datasets, we identified, among other genes, the *NAC-like transcription factor 42* [VIT_12s0028g00860] gene expression of which was apparently responsive to PM, but not to elevated levels of SA. The *NAC* transcription factor genes form a large plant-specific gene family, members of which have been implicated in development, fruit ripening, senescence, abiotic and biotic stress responses^20^,^21^,^22^,^23^,^24^,^25^. However, the activation of *NAC042_5* in the defense response, especially on the effect of PM infection in grapevine, has yet to be fully understood. Phylogenetic analysis of the coding sequence of this gene revealed that the nearest *A. thaliana* orthologue is *JUNGBRUNNEN1* (*JUB1*)/*AT2G43000*, which was also induced by *Golovinomyces orontii*-infection in *Arabidopsis*^13^. However, its expression was influenced by the *ics-1* and *sid2-2/eds16* mutations, suggesting that the expression of *Arabidopsis JUB1* may depend on SA signaling^13^,^26^. By investigating the promoter activity of the grapevine *NAC042_5* gene in transgenic *Arabidopsis,* we provide evidence that it is regulated in response to PM colonization in a SA-independent manner.

## Results and Discussion

Numerous studies have demonstrated that transcriptome remodeling induced by obligate plant pathogens is mediated to a great extent by SA signaling^27^. PM pathogens have been shown, however, to induce changes in the transcriptome well beyond SA-induced gene expression^13^. To distinguish host transcriptome changes triggered exclusively by SA from those triggered more broadly by *E. necator* colonization, we conducted two separate global leaf transcriptome analyses using the Vitis Affymetrix GeneChip platform. In the first experiment, we compared the leaves with fully established PM colonies to healthy reference leaves, and found that transcript abundance was at least 1.5-fold higher or lower for 373 genes in PM-infected leaves relative to healthy reference leaves (Supplementary Table S1). Whereas the SA was below the threshold of detection in control leaves, SA accumulated in subsamples of PM-infected leaves to 0.92 ± 0.68 μg/g fresh weight. These SA levels were similar to those measured in PM-infected grapevine leaves at 2 days post-inoculation (dpi)^10^, indicating that SA levels remained high even when PM colonies became well established on grapevine leaves. This suggests that defense signaling was active in leaves supporting mature, well established PM colonies.

In the second experiment, we assayed MeSA-treated grapevine leaves in comparison with control leaves. The total SA concentration was significantly higher in the MeSA-treated plants (26.33 ± 12.48 μg/g fresh weight) than in control leaves where SA was undetectable. We found that 481 genes responded to the MeSA treatment with at least 1.5 fold-change in expression, and 179 of them were a subset of the PM-regulated gene list. This suggests that a subset of PM-responsive genes may be regulated via SA signaling.

**Figure 1:**
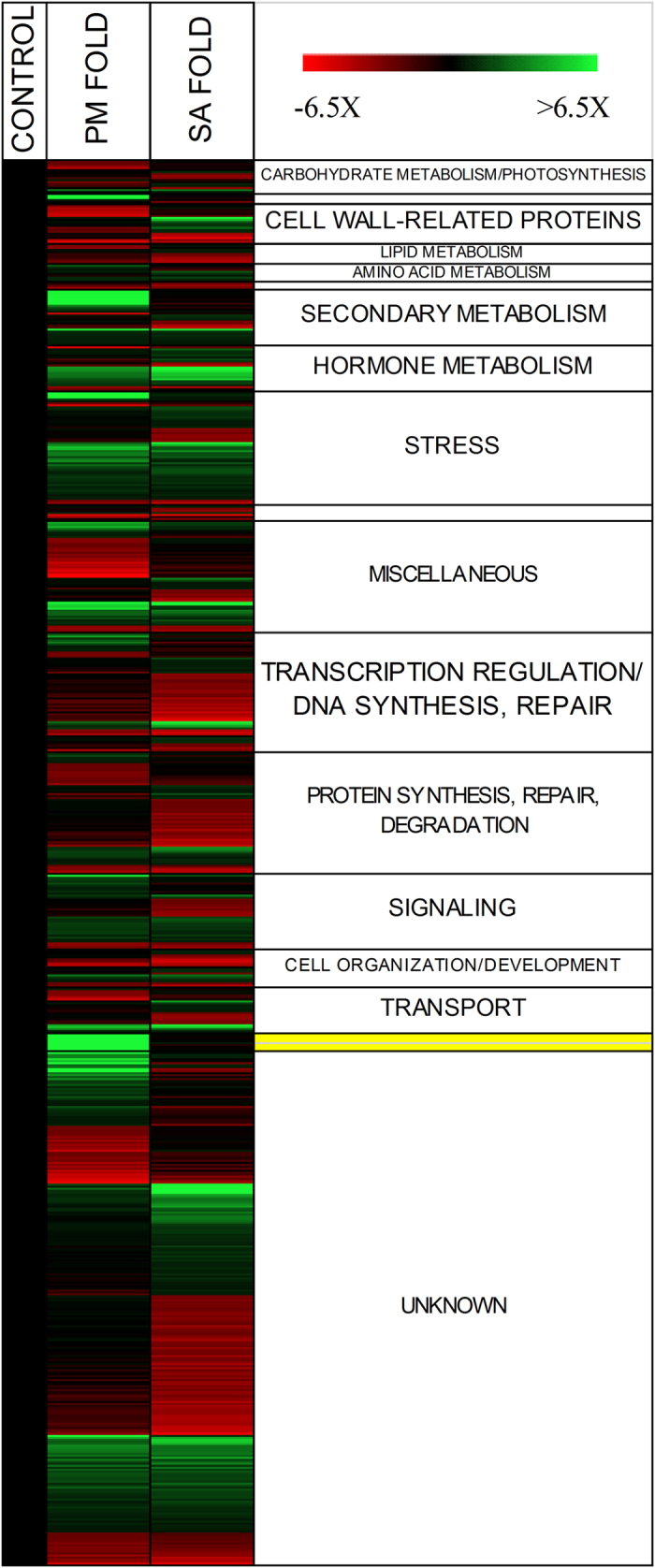
Changes in expression rate measured by microarray analysis. 675 significantly altered probe sets that were up- or down-regulated by at least 1.5-fold relative to control. The yellow color represents the reference fungal genes. Black: 1x expression; red: 6.5-fold down-regulated; green: 6.5-fold or above up-regulated.

The Vitis Affymetrix GeneChip included nine probe sets of fungal origin with a nearest homology to genes of ascomycetous fungi (Supplementary Table S1). All of these nine genes were identified in our microarray results as exclusively PM-dependent and were among the genes with highest expression rates (8- to 284-fold). The hybridization of these probe sets by transcripts in exclusively PM-treated samples confirmed that *E. necator* inoculum was absent in MeSA-treated and control samples proving that experimental treatments were carried out appropriately (Fig. 1).

**Figure 2:**
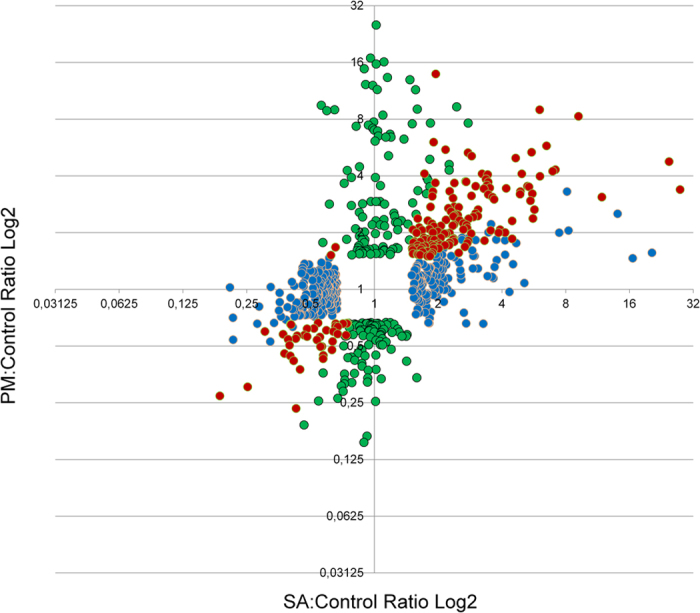
Bivariate plot of expression fold-change for genes that respond to PM colonization and/or MeSA treatment in grapevine leaves. Fold-change of expression induced by PM (vertical axis) plotted against fold-change of expression induced by MeSA (horizontal axis) for genes that respond at the 99% significance level with at least 1.5-fold up- or down-regulation allowing for 5% false discovery rate. Graph does not include data point for the fungal gene derived probe set (1615715_at). Green: PM-responsive only; blue: MeSA-responsive only; red: both treatment-responsive.

The relative transcriptional change of those genes that were found to be modulated by PM infection only, MeSA treatment only, or both PM infection and MeSA treatment are displayed in Fig. 2. The microarray probe sets and grapevine transcripts in these categories as well as their nearest *Arabidopsis* homologues are listed in Supplementary Table S1.

### Validation of microarray results

Validating the results of the microarray analysis with qPCR showed that the overall tendency of expression changes was similar to that detected by the microarray (r^2^ = 0.762) (Supplementary Data S2). *PRP1* [VIT_03s0088g00710], *Bet v I allergen* [VIT_05s0077g01540], and *NAC042_5* [VIT_12s0028g00860] were significantly up-regulated by PM, whereas a gene encoding a lipid transfer protein [VIT_04s0008g05640] was suppressed. Concerning gene regulation influenced by SA, the expression of *PRP1* increased, whereas expression of the *ADAGIO PROTEIN 1* [VIT_01s0011g05810] was suppressed. However, not all expression changes that were significant in the microarray data could be confirmed as significant changes in the qPCR analysis. For example, although the *FAH1* [VIT_07s0031g01380] was registered as up-regulated in both microarray and qPCR experiments, the change was significant (p = 0.0005) only in the microarray data.

### Genes induced by both MeSA treatment and PM colonization

Among the 179 transcripts that responded in a similar way to PM and to MeSA, we found genes that function in biotic stress signaling as well as in primary and secondary metabolism (Fig. 1). We refer to these genes as the PM- and SA-regulated gene set. The key signaling molecule for systemic acquired resistance is MeSA, a mobile form of SA^7^. Gene *SAMTBSCMT* [VIT_04s0023g02240] was found to be up-regulated by PM as well as by MeSA treatment. *SAMTBSCMT* encodes a salicylate O-methyltransferase which catalyzes the formation of MeSA from SA and regulates MeSA formation at the site of infection; MeSA is then delivered to the systemic uninfected region of the plant where it can be converted back to SA by SABP2 (SA binding protein 2) to fulfill its function^28^. We found that most of the typical defense-associated genes responded to MeSA treatment. During pathogen attack, the receptor-like protein kinases (RLKs) are the first key regulator proteins of pathogen-associated molecular patterns-triggered immunity (PTI). Among the identified kinases, many of them belong to leucine-rich repeat domain-containing RLKs, which regulate a wide variety of defense responses^29^. From the identified 25 PM-responsive RLKs, 15 were stimulated by MeSA. Three of these were homologous to the *Avr9/Cf-9 Rapidly Elicited 256* gene of tobacco, which is one of the key regulators of the HR during biotic stress^30^. Another key defense signaling gene that was found both MeSA- and PM-inducible is *Enhanced Disease Susceptibility1* (*EDS1* [VIT_17s0000g07420]). Albeit, EDS1 is an upstream regulator of SA, previous studies demonstrated that abundant SA may feedback-regulate the EDS1/PAD4 complex in *Arabidopsis*^28^. It has recently been shown that *V. vinifera EDS1* is induced in response to SA and that its orthologue from a PM-resistant *V. aestivalis* grape variety has a distinct expression pattern^31^.

Defense signaling downstream from SA is largely continued by activation of NPR1/NIM1, where NPR1 is interacting with NIMIN1, 2, 3 (NIM-interacting1, 2, 3) and several TGA factors to induce defense gene expression^32^. Although NIMIN-1 acts as a negative regulator of SA/NPR1 signaling^33^, we found a gene, probably encoding the grape orthologue of NIMIN-1 [VIT_07s0005g02070], which was up-regulated in response to both treatments. NPR1, TGA2, 3, 5, and/or 6 control *WRKY* transcription factor genes, which may positively or negatively regulate the defense response^34^. The grape orthologue of *WRKY18_2* [VIT_04s0008g05760], an *Arabidopsis* gene known to positively and negatively regulate SA/EDS1-mediated resistance against *Pseudomonas syringae* and *G. orontii,* respectively^35^, was stimulated by both treatments. In addition, we also found two Myb-type transcription factor (TF) genes, namely, *MYB108* [VIT_05s0077g00500] and *MYB14_3* [VIT_05s0049g01020], to be MeSA-inducible. The MYB108 TF belongs to the R2-R3-type MYB family, members of which are known to be involved in the SA-signaling pathway^36^. *MYB108* is closely related to the ABA-dependent *BOTRYTIS SUSCEPTIBLE1* gene, which is a negative regulator of cell death triggered by wounding or pathogen attack^37^.

The following pathogenesis-related (*PR*) genes were regulated via both SA, and PM: *PRP1* genes [VIT_03s0088g00710/VIT_03s0088g00810/VIT_03s0088g00700/VIT_00s0207g00130]*, BG3* genes [VIT_06s0061g00120/VIT_08s0007g06040], *PR-3* [VIT_03s0038g03400], *CHIV* genes [VIT_05s0094g00360/VIT_05s0094g00350/VIT_05s0094g00220], *CHIB1* [VIT_16s0050g02220], *OSM34* genes [VIT_02s0025g04250/VIT_02s0025g04330/VIT_02s0025g04340/VIT_02s0025g04310], *PRXR11* [VIT_07s0129g00360] and *NtPRp27 secretory protein* [VIT_03s0091g00160]. *PR* genes were expressed over the course of the infection process with a steady increase starting at early infection stages^10^. Due to their expression pattern, they all were allocated to the same cluster^10^. It is likely that the regulation of *PR* genes during PM infection is indicative of the coordination of the defense response via SA signaling, as it was found for other plant-pathogen interactions^26^. Fungal infection-triggered PR protein secretion may be assisted by chaperone proteins^38^. The expression of chaperone genes *calnexin 1* (*CNX1* [VIT_00s0283g00030]) and *endoplasmin* (*SHD* [VIT_18s0001g14500]) was found to be up-regulated in PM- and SA-dependent manner, as it was earlier shown for their orthologues in *Arabidopsis*^13^.

PM infection along with SA signaling may also induce cross-linking of molecules in the plant cell wall and/or deposition of lignin as part of PTI^39^, which is indicated by the enhanced expression of *OMT1* (caffeic acid O-methyltransferase [VIT_16s0098g00850]), a gene known to be involved in lignin synthesis^40^.

Genes encoding heat shock proteins (HSPs) (*HSP70-1* [VIT_08s0007g00130], *HSP17.6II* [VIT_04s0008g01490], and *BIP1* [VIT_16s0098g01580]), a heat shock-related TF (HSF4 [VIT_07s0031g00670]), a DNAJ homolog (ERDJ3B [VIT_07s0005g01220]) and an Aha1 domain-containing protein [VIT_08s0007g06710] functioning as activator of HSPs^41^ all responded to both PM and SA. HSPs are involved in abiotic stress signaling and their role in plant responses to pathogen attack has yet to be fully understood. However, these proteins were found to be also active under oxidative stress, as reactive oxygen species (ROS) and photorespiratory H_2_O_2_ induces their expression^25^,^42^. The earliest response after PM infection in *V. vinifera* is an oxidative burst, and rapid up-regulation of genes involved in protection from ROS^10^. The pathogen-triggered ROS could explain that these heat shock protein encoding genes are up-regulated by both treatments. Furthermore, low levels of H_2_O_2_ act as a signal for defense gene expression^43^, which is supported by the PM and MeSA-dependent up-regulation of a reticuline oxidase precursor transcript. Reticuline oxidase (BBE) catalyzes H_2_O_2_ production by using hexose sugars and it mediates basal resistance against pathogens^44^. However, the *MSS1* (sugar transport protein 13 [VIT_11s0016g03400]) was also up-regulated by both treatments.

Defense responses along the SA-mediated pathway include redox signaling which is based on the glutathione (GSH) and disulphite (GSSG) ratio. Glutathione S-transferases (GSTs) have both conjugase and peroxidase activity, therefore, GSTs use GSH and reduce H_2_O_2_ amount, thereby increasing GSSG levels^45^. Indeed, glutaredoxins (GRXs), namely, GRX480 [VIT_10s0003g00390] and a cytosol localized GSTU8 [VIT_08s0007g01400], which lower H_2_O_2_ and elevate GSSG levels were found to be up-regulated by both PM colonization and MeSA treatment. It has been demonstrated that AtGRX480 mediates redox regulation by TGA factors during stress, and linked to SA-dependent pathway^46^. We found in grapevine, however, that another glutaredoxin [VIT_07s0104g01390] was markedly repressed by both treatments.

Among the PM-responsive ATP binding cassette (ABC) transporters, we identified three genes, which were up-regulated by MeSA. One identified transporter probably belongs to the C, the two others to the G family (ABCG7 [VIT_00s0625g00020/VIT_03s0017g01280]). Notably, the expression of the G family members were induced to very high levels by MeSA (6- and 25-fold). Members of the G family are known to mediate the export of cuticular lipids, with *PEN3* being a key player in the defense response in *Arabidopsis*^47^,^48^.

We found several PM-stimulated secondary metabolism-related genes which play a role in the biosynthesis of antimicrobial compounds. The genes encoding HMG-CoA-synthase (*MVA1* [VIT_02s0025g04580]) and HMG-CoA –reductase (*HMGR1* [VIT_03s0038g04100]) were activated by both PM and MeSA treatments. The MVA1 and HMGR1 proteins are components of the isoprenoid biosynthesis pathway and involved in the synthesis of mevalonate^49^. Mevalonate is the precursor of phytosterols which play a key role in innate immunity and restrict the nutrient efflux into the apoplastic space where nutrients may be taken up by the pathogen^50^. Moreover, it has been demonstrated previously that the over-expression of *Brassica juncea HMG-CoA-Synthase1* in *Arabidopsis* resulted in the constitutive expression of *PRP1*, *PR2* and *PR5* along with suppression of H_2_O_2_-induced cell death^51^, which is in agreement with our findings in grapevine presented here.

Genes involved in aromatic amino acid and phenylpropanoid biosynthetic pathways, such as *prephenate dehydratase* (*PD1* [VIT_06s0061g01300]), *anthocyanidin O-glucosyltransferase* (*RHGT1* [VIT_16s0050g01680] and *GT* [VIT_03s0017g02110/VIT_12s0034g00130]), *UGT89B1* [VIT_17s0000g04750] and *DMR6* genes [VIT_16s0098g00860/VIT_13s0047g00210] were found to be inducible by both MeSA and PM. This is concordant with the notion that flavonoids and their anthocyanin derivatives, have anti-fungal activity in grape varieties^52^. However, the *Arabidopsis AtDMR6* gene was found to provide susceptibility to downy mildew^53^. Transcription of the flavonoid biosynthetic gene, *CYP706A4* (encoding flavonoid 3′-hydroxylase [VIT_00s1682g00020]) as well as the cytokinin glucosyltransferase gene, *UGT85A2* [VIT_00s0324g00070] were down-regulated by both treatments.

SA antagonizes JA signaling in various biotic stresses, and it was found that increased SA levels along with repression of JA-signaling resulted in resistance against biotrophic pathogens, but provided susceptibility to necrotrophs^54^. This cross-talk may be partially dependent on the cellular redox status, while overexpression of *GRX480* induced *PR-1*, but repressed *PDF1.2*^55^. Confirming this relationship, MeSA as well as PM, induced the expression of *JAZ1_2* [VIT_09s0002g00890] in our study. JAZ proteins were shown to repress transcription of JA-responsive genes^56^. However, synergism was also observed between these two signaling pathways as SA signaling does not always repress JA biosynthesis^57^. We found three genes, *LOX2* (lipoxygenase [VIT_06s0004g01510]), *CYP74A* (allene oxide synthase [VIT_18s0001g11630]), and *OPR2* (12-oxophytodienoate reductase 2 [VIT_18s0041g02020]) participating in JA synthesis, which were up-regulated in response to both treatments. This is consistent with a recent study which demonstrated that *LOX* expression in cucumber was stimulated not only by PM and SA, but also by JA and ABA^58^.

The basal defense of susceptible plants also implicates processes that lead to cell wall fortification in response to pathogen attack. We identified two cell wall-related genes, *EXPA8* [VIT_13s0067g02930] and a pectate lyase [VIT_17s0000g09810], which were down-regulated by both treatments. Expansins unlock the network of wall polysaccharides and pectate lyases degrade the pectin component of cell wall^59^,^60^, therefore, their repression maintains cell wall integrity. PM-induced repression of these grapevine genes via SA-signaling suggests a regulation by the plant to boost structural resistance against the invading pathogen. In *Arabidopsis*, down-regulation of the pectate lyase-like gene *PMR6* was shown to enhance resistance to PM^61^. Thus, PM-induced repression of these grapevine genes suggests that their down-regulation may also contribute to enhanced resistance.

Overall, expression of most genes modulated by both MeSA and PM were part of the SA-mediated defense response The majority of the MeSA- and PM-responsive transcripts are downstream of SA in the signaling cascade (as the NIMIN1-1, WRKY or PR proteins), but some upstream regulators (EDS1) are also known to participate in a feedback-regulatory loop with SA.

### Genes induced by PM colonization but not by SA treatment

Among the PM-regulated genes in grapevine, 185 candidates were identified which were not triggered solely by MeSA, indicating that elevated SA levels alone cannot substitute for regulation by PM. These 185 genes are referred to as the “PM-dependent” gene set. These include numerous genes that are involved in primary metabolism, including the pathways of carbohydrate, protein, and fatty acid metabolism (Fig. 1, Supplementary Table S1). Since PMs are obligate biotrophic pathogens, they must rely on their host as carbon and nitrogen source and, therefore, modulate plant metabolic processes to fulfill their needs. However, previous results demonstrated that carbohydrates also may have signaling function in defense responses as the increased content of soluble sugar induced the expression of *PR* genes in *Arabidopsis*^62^. Beside the activation of defense-genes, sugar accumulation is also expected to decrease photosynthesis^63^. In agreement with these expectations, we found that all photosynthesis-related PM-dependent genes, including photosystem II 22 kDa protein [VIT_18s0001g02740], photosystem II light harvesting complex 2.1 [VIT_12s0057g00630], NADH dehydrogenase I subunit N [VIT_06s0004g08360], plastocyanin-domain containing protein [VIT_02s0025g02410], LHCII-type I CAB-1 [VIT_19s0014g00160], and light-harvesting chlorophyll-binding protein 3 [VIT_00s0181g00200], were down-regulated in response to PM infection. Potentially, the down-regulation of these genes could be linked to plant defense responses. For example, PM infection induced the expression of *MES17* pheophorbidase gene [VIT_13s0067g03260) which may participate in chlorophyll breakdown^64^, a consequence of programmed cell death.

An early response to pathogen infection is the apoplastic accumulation of ROS, which may be mediated by aquaporins. However, PM infection repressed *AQUAPORIN TIP1_3* [VIT_06s0061g00730] encoding a protein known to translocate H_2_O_2_ across the plasma membrane^65^. Interestingly, RNAi silenced *tip1-1 Arabidopsis* plants revealed an increased apoplastic carbohydrate content^66^, suggesting that *AQUAPORIN TIP1_3* suppression in infected grapevine may support the sugar availability for the pathogen. In addition, the transcription of a germin-like protein-encoding gene [VIT_17s0000g05360] was also induced by PM. Such proteins were found to catalyze H_2_O_2_ production^67^.

Among the PM-dependent gene set, several transcription factors were identified, among them a *NAC*-type transcription factor (*NAC042_5* [VIT_12s0028g00860]). Based on the expression pattern reported earlier^10^, this gene belongs to the same cluster as genes for pinoresinol forming dirigent protein (*DIRPR* [VIT_02s0025g00750]), dicyanin blue copper protein (*BCB* [VIT_09s0002g06890]) and isoflavone methyltransferase [VIT_12s0028g01940]^10^. These latter genes were also PM-dependent, albeit their cluster also contains *PR* genes^10^ which were PM and MeSA-inducible in our current dataset. Two other transcription factors that belong to the WRKY family (*WRKY71_2* [VIT_12s0028g00270] and *WRKY21_2* [VIT_00s2547g00010]) were in the PM-dependent gene set. Previous studies demonstrated that WRKY71 is involved in the defense response and that it is an upstream regulator of NPR1 in rice^68^. The WRKY IId subfamily members, including WRKY21, were found to interact with Ca^2+^/calmodulin binding transcription factors^69^ and mediate the defense response. However, the transcription of a calmodulin-binding protein [VIT_01s0026g01790] was found to be up-regulated by both MeSA and PM.

Two typical defense associated genes, namely *PR10* [VIT_05s0077g01530] and *Bet v I allergen* [VIT_05s0077g01540], were strongly expressed (8- and 13-fold up-regulation) only in response to PM. Although most PR transcripts were found to be MeSA-inducible, these genes responded only to PM. They were grouped in a cluster along with genes encoding stilbene synthases and the cytochrome P450 84A1 (FAH1)^10^. Several studies proved that PR-10 proteins, which have RNase, DNase, and anti-fungal activity, play a role in defense responses and cell death and that they are regulated by WRKY TFs^70^,^71^. It was shown that the expression of the *V. vinifera PR10.1* was transcriptionally regulated by the WRKY33 TF due to *Plasmopara viticola* infection^72^. Further, it was demonstrated that the *Asparagus PR10* was responding to pathogen infection and H_2_O_2_ independently from SA^73^, which suggests that these proteins mediate defense responses upstream or independent of SA signaling in grapevine also.

Some genes involved in the biosynthesis of stilbenoids, flavonoids and phenylpropanoids were found to be regulated in a PM-dependent manner. The genes encoding DAHP- and EPSP-synthases [VIT_00s0391g00070 and VIT_15s0048g00350] were found to be up-regulated. The corresponding proteins catalyze the synthesis of aromatic amino acids, which are precursors of flavonoids and stilbenoids in the shikimate pathway^74^. The expression of stilbene synthase genes (*STS2* [VIT_16s0100g00990], *STS4* [VIT_16s0100g01000], *TT4* genes [VIT_16s0100g01190/VIT_16s0100g01140/VIT_16s0100g00840]), as well as the expression of an R2R3-type MYB factor gene [*MYB14_2* [VIT_07s0005g03340] which likely regulates stilbene biosynthesis, was 3- to 7-fold up-regulated by PM in grapevine. Previously, a *MYB14* was found to be co-expressed with *STSs* and to specifically interact with the promoters of *STS41* and *STS29* in grapevine^75^. Stilbenes in *Vitis* species were proposed to be part of the plant arsenal against *E. necator*^76^. The gene, encoding chalcone-flavonone isomerase (*TT5* [VIT_13s0067g03820]) involved in flavonoid biosynthesis was also regulated in a PM-dependent manner. A similar response was found for a putative *DFRA* [VIT_08s0040g00440] and a *UGT75C1* [VIT_05s0062g00740] gene, which are involved in secondary metabolism. UGTs along with cytochrome P450 monooxygenases play a key role in creating the structural diversity of triterpenoid saponins^77^, which are antifungal compounds^78^. Among the six PM-dependent cytochrome P450 genes identified, four were up-regulated (*FAH1* genes [VIT_07s0031g01380/VIT_04s0023g02900], *CYP87A2* [VIT_02s0025g04080], *CYP716A1* [VIT_11s0065g00130]) and two were down-regulated (*CYP714A1* [VIT_13s0067g00110], *CYP87A2* [VIT_02s0025g04080]) in response to the presence of pathogen. Corroborating our findings, the *Arabidopsis FAH1* was also found to be up-regulated by PM independently of SA signaling^13^. The cytochrome P450 gene *CYP716A1*, protein product of which is involved in antimicrobial saponin biosynthesis, was up-regulated 9-fold in response to PM, supporting the premise that it mediates plant defense^79^. Geraniol 10-hydroxylase [VIT_15s0048g01490] is involved in terpenoid indole alkaloid biosynthesis^80^, and its gene is homologous to *AtCYP76C1*, which was also down-regulated independently from SA in response to PM in *Arabidopsis*^13^. Since these genes were not inducible by SA, the inducer is likely to be another signal. *CYP87A3* (61% identity to *CYP87A2*) was previously reported to be responsive to auxin^81^ which may also act as a defense signal during pathogen attack and it may have an antagonistic regulatory role to SA^82^.

Four of the six PM-responsive dirigent-like protein genes [VIT_06s0004g01020/VIT_02s0025g00750/VIT_06s0004g01010/VIT_06s0004g00990], which play a role in lignin synthesis^83^ were strongly up-regulated (3- to 13-fold) in a PM-dependent manner, which is in agreement with previous reports^84^. Another lignin biosynthetic gene encoding a cinnamoyl-CoA reductase [VIT_02s0012g01570], was found to be regulated by PM only. The protein product of this gene promotes the H-, S-, and G-lignin formation in the monolignol pathway.

The acyl-CoA-binding domain 3 proteins (ACBP3) are proposed to be involved in lipid metabolism. However, the *Arabidopsis* ACBP3 also regulates the NPR1-dependent defense in response to the biotrophic bacterium *P. syringae*, and overexpression of *ACBP3* resulted in enhanced *PR* expression, cell death and H_2_O_2_ production^85^. We found that the *ACBP3* [VIT_07s0129g00430] grapevine gene is not MeSA-inducible, but it is triggered by the pathogen. In contrast, other genes encoding enzymes involved in the lipid metabolism (3-oxoacyl-[ACP] reductase [VIT_01s0010g02670], a probable sulfotransferase [not registered in Ensembl]) and in lipid transfer/binding [VIT_04s0008g05640/VIT_11s0016g05840/VIT_04s0008g05640] were at least 6-fold down-regulated by PM, in accordance with previously reported results^10^. Since lipids have a signaling function during pathogen attack, the PM-mediated down-regulation of the expression of such genes may halt activation of defense responses.

### Observation of the *NAC042_5* promoter regulation

Among the most dramatically regulated PM-dependent genes there was *NAC042_5* which codes for NAC-like transcription factor 42. The expression of *NAC042_5* was induced 7-fold in response to the PM fungus, but was unchanged in response to MeSA treatment (Supplementary Table S1), suggesting PM-specific and SA-independent regulation. To confirm that the transcription of *NAC042_5* was indeed SA-independent, a *pNAC042_5::GUS* translational fusion reporter was constructed and inserted in the genome of wild-type *A. thaliana* Wassilewskija (WS-0), SA-signaling mutant WS-*nim1-1*, and SA-deficient transgenic WS-*nahG* plants. In the designation of this construct, *pNAC042_5* denotes a 3896 bp-long stretch sequence of the *pNAC042_5* promoter (National Center for Biotechnology Information GenBank accession number: KU297673) and the first 8 amino acids of the NAC042_5 polypeptide.

**Figure 3:**
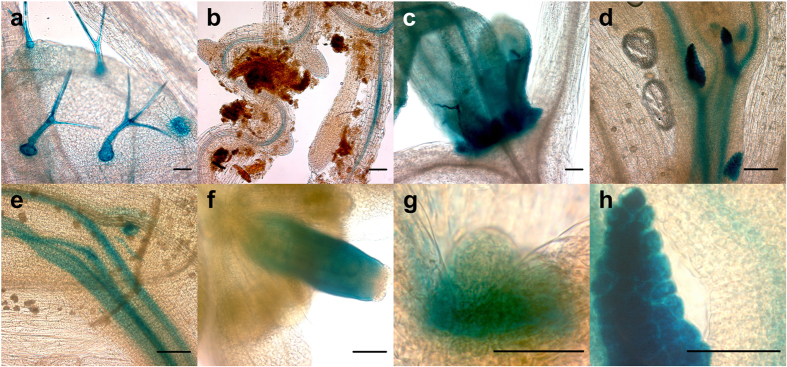
Tissue-specific regulation of the *NAC042_5* promoter in transgenic *Arabidopsis* plants. (**a**) Leaf hairs, (**b**) roots, (**c**) developing new leaves, (**d**) lateral shoot buds of developing inflorescence, (**e**) vascular tissue, (**f**) developing silique, (**g**) shoot apical meristem, (**h**) shoot bud. (**a,b,d–f,h**) three week-old plants, (**c**) two week-old plant, (**g**) five day-old seedling. Length of scale bars correspond to 50 μm.

GUS staining of non-inoculated homozygous transgenic plants demonstrated that all three types of transgenic *Arabidopsis* (WS-0, WS-*nim1-1*, and WS-*nahG*) showed a similar basal GUS expression independent of PM challenge. Earlier studies demonstrated that members of the *Vitis NAC* gene family regulate organ development in grapevine species, and that their expression differed in various developmental stages^86^,^87^. The expression of *NAC042_5* did not demonstrate strict tissue-specificity, as its promoter was active in the shoot apical meristem, young developing shoots and leaves, siliques, trichomes, vascular tissues, and lateral shoot buds (Fig. 3). Promoter activity in this broad variety of organs could be explained by a more general transcriptional regulator function.

### *NAC042_5* promoter activity in response to PM infection

**Figure 4:**
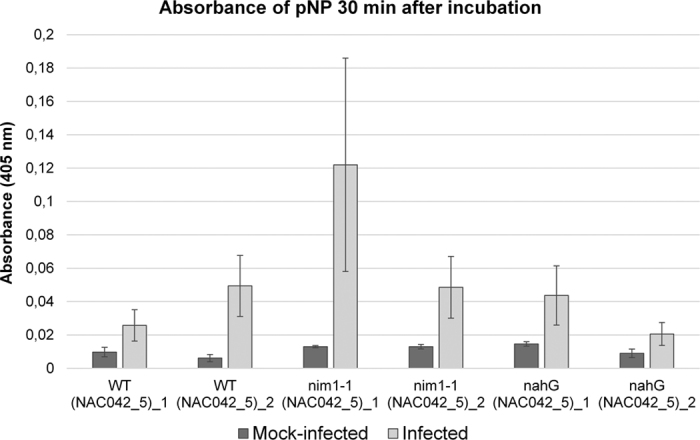
Response of the *NAC042_5* promoter to PM infection in transgenic *Arabidopsis*. GUS quantification based on *pNAC042_5::GUS* activity due to 14 day-old PM colonies on leaves of transgenic *Arabidopsis* lines of WT, *nim1-1*, and *nahG* genetic backgrounds. Columns represent independent lines; each of them with at least three biological repeats; error bars represent the standard error.

To quantify PM-induced transcriptional activity directed by the *NAC042_5* promoter, we inoculated and mock-inoculated the *pNAC042_5::GUS* transgenic *Arabidopsis* lines with *Oidium neolycopersici* following the method described by Huibers and co-workers^88^. By 14 dpi, the inoculation led to fully developed conidium-producing PM colonies in all lines, and all mock-treated plants remained PM-free. PM infection advanced faster and produced more extensive colonies in plants of the *nim1-1* and the *nahG* genetic background than in wild-type plants, which is likely due to the higher disease-susceptibility of *nim1-1* and *nahG* plants. Leaf tissues with 14 day-old PM colonies and mock-inoculated control leaves were used for a *p*NPG spectrophotometric assay to quantify GUS activity. Statistical analysis of the GUS assay data revealed that the interaction effect of treatment and assay time is significant (*p* < 0.0001), and this significance of the PM-infection occurred at 0 and 30 min. At subsequent time points during the spectrophotometric assay, the variability of absorbance values increased with time and the absorbance values were also correlated in the PM-infected samples. After adjusting for the dependence and the varying variability, the estimated rate of change is 1.34 times of the median, which is significant. The confidence limit for the rate is 1.112 and 1.568 times of the median. We also detected a marginally significant (*p* = 0.0485) effect of the interaction between the treatment and genetic background which probably reflects the more intense growth of the PM pathogen in the highly susceptible *nim1-1* and *nahG* lines than in the wild-type^89^. Values from plants of independent lines for each type of transgenic plant with a similar basal expression are displayed (Fig. 4). As SA signaling is abrogated in *nim1-1* and *nahG* plants, these results provide evidence that the *NAC042_5* promoter is responsive to PM infection in an SA-independent manner. Two recent studies presented that another *Vitis* NAC transcription factor gene, *NAC1*, was activated by *E. necator* along with increased expression of defense-associated genes, such as *PDF1.2, VSP1, PR1, PR2 PR4* and *PR5*^86^,^90^. However, in contrast to the *NAC042_5* used for our investigations, the *NAC1* gene was found to be SA-inducible, indicating that the expression of the various grapevine *NAC* genes is regulated by different signaling pathways.

**Figure 5:**
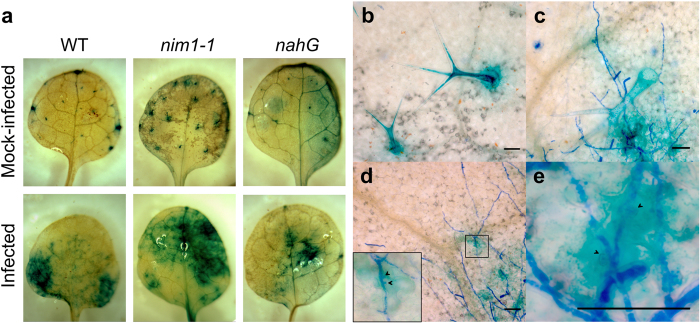
Histochemical staining of *pNAC042_5::GUS*-transgenic plants following *O. neolycopersici* inoculation. (**a**) GUS-staining of mock-inoculated control leaves and *O. neolycopersici*-infected leaves (second rosette leaf) at 11 dpi, (**b**) microscopic image of mock-inoculated leaf, (**c–e**) microscopic images of cotton blue-stained PM hyphae (dark blue) on GUS-stained leaf tissue after inoculation. Note the intense *GUS* staining (light bue) visible in the trichome (**b,c**) and along the PM hyphea (**c–e**). Inset on picture (**d**) is an enlargement of an infected epidermis pavement cell. Arrowheads point to fungal haustoria. Length of scale bar corresponds to 50 μm.

The *pNAC042_5::GUS* reporter lines were also investigated by histochemical staining in response to PM infection and the staining of these leaves revealed a marked increase in *GUS* activity at the sites where PM colonies developed (Fig. 5a). In mock-inoculated control leaves, GUS-staining was mostly limited to trichomes (Fig. 5b). To confirm that GUS-staining was indeed caused by the growth of *O. neolycopersici* colonies, we also stained the fungus with the dye cotton blue. Robust GUS-staining was always associated with the presence fungal structures (Fig. 5c–e) and never occurred in their absence. On mock-inoculated leaves, only few confined GUS spots were visible, but this was clearly distinguishable from the robust GUS-staining detected at fungal infection sites (Fig. 5a,c,d). This indicates that the reporter gene was strongly expressed in only those areas of the leaf where the pathogen had direct contact with the plant tissue (Fig. 5c–e). Higher magnification revealed that *GUS* expression severely increased mostly in those cells, in which the fungus developed haustoria (Fig. 5d,e). This PM-dependent increase in *GUS* activity was found in all three types of transgenic plants (with *nim1-1*, *nahG*, and WT background), which provides further evidence that *NAC042_5* expression does not require SA signaling.

The *Arabidopsis* gene *ANAC042/JUB1,* the ortholog of the grape *NAC042_5*, was also shown to be up-regulated exclusively in the immediate vicinity of cells invaded by haustoria in the *G. orontii-Arabidopsis* interaction^91^. The same study found that *PR-1* expression was 137-fold up-regulated in cells closely associated with the haustorium^91^. Furthermore, the ABC transporter gene *PEN3*, which mediates penetration resistance, also showed infection site-specific transcription^47^. Similarly, *PUX2* and *DMR6*, genes which support mildew development in *Arabidopsis*, were also up-regulated at the site of infection^53^,^91^. Additional examples of SA-independent PM-responsive genes are *PMR5* and *PMR6*, which are required for the accommodation of the fungal haustorium at later stages colonization^12^,^61^.

## Conclusions

In grapevine, PM colonization triggers changes in expression of a broad range of genes, many of which were not responsive to an increase in SA levels alone. This suggests that PM colonization activates regulatory networks that are more extensive than the SA-mediated defense system. Furthermore, genes with no known defense-related function have also been observed to change in expression. One of these is an NAC-type transcription factor gene (*NAC042_5*). The cloned promoter of this gene was activated in tissues colonized by the PM fungus *O. neolycopersici* in *nim1-1*-mutant and *nahG*-transgenic *A. thaliana* lines which are SA signaling-impaired and SA-deficient, respectively. These results provide experimental evidence that PM colonization activates regulatory mechanisms that are independent of SA-mediated regulation.

## Methods

### Grapevine plant material, growth conditions, and PM-/MeSA-treatments

The experiments were performed with one-year-old greenhouse-cultivated potted *V. vinifera* L. cv. ‘Cabernet Sauvignon’ grapevines with a single actively growing herbaceous shoot on each vine. To prepare PM-colonized tissues, two unfolded, but still expanding leaves were mock-inoculated or inoculated with *E. necator* conidia under greenhouse conditions. Inoculation was done by touching the upper surface of the leaf with a detached grapevine leaf covered with *E. necator* colonies actively producing conidia. To prepare leaf tissues for SA-induction, leaves at the same developmental stages were mock-inoculated by touching the leaves with detached PM-free healthy grapevine leaves. To prepare healthy reference leaf tissues, plants were treated in the same manner, including mock-inoculation. Three dpi, all grapevines were transferred to a PGR15 plant growth chamber (Conviron) with conditions of 85% RH, 14/10 h diurnal cycle, and 26 °C temperature. PM-inoculated plants were cultivated in the growth chamber for eight additional days until 11 dpi, at which time the PM-colonized leaves were harvested for RNA extraction. Plants prepared for SA-induction were cultivated in the growth chamber for seven days, at which time they were treated with 15 μM of methyl salicylate (MeSA, SA analogue), evaporated in the atmosphere of the growth chamber under airflow generated by a computer fan for 24 hours. SA-induced mock-inoculated leaves were harvested at the completion of this 24-hour treatment (11 dpi). Reference plants were cultivated under identical growth chamber conditions with their mock-inoculated leaves harvested at 11 dpi. Thus, the PM-colonized and reference samples differed only in the presence/absence of PM treatment, whereas the SA-induced and reference samples differed only in the presence/absence of MeSA treatment. Leaves from all treatments were harvested at 11 dpi and immediately flash-frozen in liquid nitrogen. Each treatment was done in three biological repeats, that is, each experiment was repeated three times in 14-day intervals with dedicated biological material. Each repeat consisted of ten potted vines. For RNA extraction, two young leaves were harvested from each vine of the ten-vine repeat and pooled into a single sample.

### Measurement of SA concentration

For SA concentration measurements, the method described by Fung and co-workers^10^ was applied. Leaf subsamples were vacuum-dried and 0.5 g of the sample was extracted and suspended in 300 μl of 20% methanol. Five microliters of the sample were used for analysis with the Agilent HPLC 1100 Series instrument with diode array detector (4.6 × 75 mm Zorbax SB-C18 3.5 μm and Zorbax High Pressure Reliance Cartridge Guard Columns, Agilent). The flow rate was 1.2 ml/min and three technical replicates were analyzed for each sample. The reference curve consisted of a dilution series of sodium salicylate in a concentration range between 1 to 100 ng/μl.

### Total RNA extraction from grapevine leaves

Leaf tissues were ground in liquid nitrogen and homogenized in extraction buffer (2% Hexadecyltrimethyl Ammonium Bromide/CTAB, 1% SDS, 2.5 M NaCl, 0.5 M Tris, 50 mM EDTA, 5% beta-mercaptoethanol, and 3% polyvinyl poly-pyrrolidone). The samples were stored at -80 °C until processing. For RNA isolation the frozen samples were thawed at 45 °C and centrifuged (13,000 rpm, 20 min, 4 °C). The supernatant was replenished with 1/2 volume of chloroform, vortexed, and centrifuged (13,000 rpm, 15 min, 4 °C). The supernatant was supplemented with 1/5 volume of 12 M LiCl and incubated for 2 hours at 4 °C. After centrifugation (13,000 rpm, 30 min, 4 °C) the supernatant was discarded and the pellet was washed twice with 80% ethanol and dissolved in RNase-free water. The samples were treated with 1 μl Turbo DNase I (Ambion) in 40 μl reactions, and RNA was purified using an RNeasy MiniElute Cleanup column (Qiagen) following the manufacturers’ guidelines.

### Microarray experiment

To analyze gene expression changes in response to PM colonization and SA, we employed the Affymetrix GeneChip *V. vinifera* (Grape) Genome Array following the manufacturer’s guidelines. Briefly, 4 μg total RNA was used to synthesize double stranded cDNA using the One Cycle cDNA Synthesis kit, then this cDNA was used to produce biotin-labeled cRNA through an *in vitro* transcription (IVT) reaction. The labeled cRNA was fragmented (heated at 94 °C for 35 min to break RNA molecules to 35- to 200-nucleotide fragments) before hybridization to Genechip probes. Hybridization was performed at 45 °C for 16 hours, followed by a washing and staining process of the array, which was performed on an Affymterix Fluidic Station 450. Fluorescence was amplified with streptavidin-phycoerythrin staining, followed by the addition of a biotinylated antibody (anti-streptavidin) solution, and by a final streptavidin-phycoerythrin staining. The prepared chip was then scanned by a GSC3000 laser scanner and the intensity values were processed using the GeneChip Operating Software version 1.2 of Affymetrix. Following Affymetrix guidelines, we performed background corrections and calculated expression values. Normalization was performed using the robust multiarray averaging method. Normalized intensity values, as well as raw GeneChip images have been deposited in the Gene Expression Omnibus database in GenBank (accession number: GSE53824).

The Affymetrix GeneChip contained nine homologous probe sets derived from ascomycetous fungi with close DNA sequence homology to *Blumeria*, *Marssonina*, and *Ajellomyces* species which served as internal controls in our inoculation experiments.

### Statistical analysis of microarray results

Intensity values of the microarray experiment were log2-transformed and submitted to exploratory analysis. An ANOVA model with balanced single factor was applied for evaluating data using the statistical package S-plus. The error term is assumed to be normally distributed with mean zero and constant variance. The genes with at least 1.5-fold change compared to the control (*p*-value < 0.01 and False Discovery Rate 5%) were selected for further analysis.

### Annotation of Affymetrix probe sets

The probes which showed at least 1.5 fold-change compared to the control were annotated. The annotation of probes was performed by blasting the Affymetrix GeneIDs (downloaded from http://www.affymetrix.com/estore/) to the EST (Expressed Sequence Tags) database of NCBI GeneBank (GeneBank and GeneIndex IDs), and the most analogous ESTs were searched for homologs among five species (*V. vinifera*, *A. thaliana*, *A. lyrata*, *S. tuberosum*, *S. lycopersicum*) using the BLASTx algorithm (Supplementary Table S1). The homology between query and database sequences was perceived to be informative only if the *E*-value was less than 1e^−10^. The identified transcripts then were analyzed by MapMan, KEGG and Ensembl databases^92^,^93^,^94^ to categorize the role of genes in metabolic pathways or other processes (Supplementary Table S1).

### Reverse transcription-quantitative real-time PCR analysis of selected genes

Selected genes were independently validated by quantitative real-time PCR (qPCR) to evaluate expression changes detected by the microarray experiment. After RNA isolation, cDNA was synthesized using the Taqman Reverse Transcription Reagent kit (Life Technologies) following the manufacturer recommendations. Based on the DFCI EST database and on the reference genome sequence provided by Genoscope (http://www.genoscope.cns.fr), gene-specific primers were designed for the following grapevine target genes (Genes and primers in Supplementary Data S2): *NAC042_5*; *PR* genes *PRP1* and *Bet v I allergen*; *FAH1*; *LTP* (lipid transfer protein); *EXPA1* (expansin A1 [VIT_14s0108g01020]); and *ADAGIO PROTEIN 1* (*FKF1*); the *ACTIN 1* served as a reference. For qPCR analysis, the SYBR Green Reagent kit (Life Technologies) and the real-time thermal cycler Mx3005P (Stratagene) were used. All samples were run in triplicates under identical reaction settings: the initial activation step of AmpliTaq Gold^®^ was 95 °C for 10 min and followed by 40 cycles with denaturation for 15 s at 95 °C, primer annealing for 30 s at T_m_ = 60 °C, and after cycling, a final segment was applied with denaturation for 1 min at 95 °C, 30 s at 60 °C and 30 s at 95 °C again. Subsequently, a melting curve with temperature steps of 1K was performed. Primer efficiency was confirmed to be similar (09 +/−01) for all primer pairs and relative quantitation was calculated using the qPCR analysis software package MxPro-Mx3005P version 3.0 (Stratagene) and the DART-PCR version 1.0 software tool^95^ as recommended. R_0_ values of target genes were normalized to R_0_ values of the reference gene. Statistical significance was determined by Student’s *t*-test to compare the treatment-induced response to the control.

### Construction of *pNAC042_5::GUS* transgenic *Arabidopsis* and inoculation with PM

Based on the grape reference genome sequence, the *NAC042_5* promoter region was isolated using the primers 5′-*CACC*TCA ATC ACA CTC AAA AAC CA-3′ (Forward) and 5′-AGT GCT AGT CTT CTC CAC CTC CAT-3′ (Reverse). The amplified DNA fragment (National Center for Biotechnology Information GenBank accession number: KU297673) was cloned into the pGWB633 binary vector^96^ using the pENTR vector system and the Gateway^®^ Cloning technology following the guidelines of Invitrogen^TM^ Life Technologies. In the pGWB633 binary construct, which was confirmed by sequencing, the *NAC042_5* promoter controls the GUS reporter gene. The T-DNA of the pGWB633 contains the *bar* gene for selection of positive transformants. The pGWB633 plasmid with the *NAC042_5* promoter construct was transferred into *Agrobacterium tumefaciens* GV3101 (pMP90) strain and, subsequently, the bacteria were used for transformation of *A. thaliana* via the flower dip method^97^. Three *Arabidopsis* lines with Wassilewskija ecotype background were selected for transformation: wild type (WS-0), *nim1-1* mutant (SA signaling deficiency, *non-inducible immunity* mutant), and *nahG* transgenic (lack of SA signal, it contains the salicylate hydroxylase gene from *Pseudomonas putida*)^89^,^98^. Following transformation, the seeds that developed from dipped flowers were harvested and sowed to grow the T1 generation under the following growth conditions: cool white light illumination, 16/8 h diurnal cycle at 24 °C degree. For selection of positive transgenic plants, we applied a solution of 60 mg/l glufosinate-ammonium containing herbicide and 0.01% Silvet L-77 on soil grown seedlings at ten to eleven days after germination^96^. The application was repeated three times until herbicide-sensitive and resistant plants could be unambiguously differentiated. The glufosinate-ammonium-resistant T1 plants were allowed to self-pollinate and produce a T2 generation. T2 seedlings were also treated with herbicide to look for the homozygous lines. The resulting plants were allowed to produce T3 progeny, which were used for inoculation experiments. Three week-old plants were mock-inoculated or inoculated with *O. neolycopersici* conidia under growth chamber conditions. Inoculation was done by touching the upper surface of the leaf with a detached tomato leaf covered with *O. neolycopersici* colonies actively producing conidia. This inoculation method was performed for histochemical assays.

For spectrophotometric measurements, four week-old plants were inoculated by spraying a conidial suspension^88^. Control treatment of plants was accomplished by a mock-inoculum spray using healthy tomato leaves. The mock-inoculated and inoculated plants were cultivated under the following growth conditions: cool white light illumination, 16/8 h diurnal cycle at 24 °C degree.

### *p*NPG measurements to quantify promoter activity in *Arabidopsis*

At 14 dpi, six individuals were collected from each line of the PM-inoculated and mock-treated plants. The infected leaf tissues were excised and were ground in extraction buffer (50 mM NaPO_4_ pH 7.0, 10 mM β-mercaptoethanol, 0.1% Triton X-100). The extract was incubated after addition of 1 mM 4-nitrophenyl β-D-glucuronide (*p*NPG) at 37 °C for 2 h^99^,^100^ and its conversion by β-glucuronidase was measured in a spectrophotometric assay at 405 nm at 30-min intervals (as repeated measurements) using a Nanodrop 1000 instrument^99^. To determine if GUS activity was different between the PM-inoculated and mock-treated tissues, the absorbance values were transformed to natural logarithm values (to obtain a reasonably normal distribution), and analyzed using a mixed linear model implemented by the software package SAS. The mixed linear model was as follows: Log (observation) = effect of gene + effect of treatment + effect of time + interaction effect of gene and treatment + interaction effect of gene and time + interaction effect of treatment and time + interaction effect of gene, treatment and time + error.

### Histochemical GUS assay to localize GUS expression in *Arabidopsis* leaf tissue

At 11 dpi, the plants were investigated by histochemical GUS assay^101^. The leaves were incubated overnight at 37 °C in the assay solution (100 mM NaPO_4_ buffer; pH 7.0, 10 mM EDTA, 1% Triton X-100, 0.3% H_2_O_2_, 0.5 mg/ml X-Gluc/5-bromo-4-chloro-3-indolyl-β-D-glucuronic acid cyclohexylammonium salt) and the chlorophyll from the samples was removed by repeatedly washing with 70% ethanol. To stain fungal tissue, leaves were dipped for 30 s into cotton blue solution (Thermo Scientific; 30X dilution in 70% ethanol), rinsed with distilled water, and subsequently investigated using a stereo- and light-microscope.

## Additional Information

**How to cite this article**: Toth, Z. *et al*. Expression of a Grapevine NAC Transcription Factor Gene Is Induced in Response to Powdery Mildew Colonization in Salicylic Acid-Independent Manner. *Sci. Rep.* **6**, 30825; doi: 10.1038/srep30825 (2016).

## Supplementary Material

Supplementary Dataset 1

Supplementary Information
