# Supplementary material for: Expression of a Grapevine NAC Transcription Factor Gene Is Induced in Response to Powdery Mildew Colonization in Salicylic Acid-Independent Manner
Source: Sci Rep. 2016 Aug 4;6:30825. doi: 10.1038/srep30825 (PMC4973223; doi:10.1038/srep30825)
Supplement: Supplementary Information [file srep30825-s2.pdf]

Expression of a Grapevine NAC Transcription Factor Gene Is Induced in Response to Powdery Mildew Colonization in Salicylic Acid-Independent Manner

Zsofia Toth, Patrick Winterhagen, Balazs Kalapos, Yingcai Su, Laszlo Kovacs, and Erzsebet Kiss

Supplementary Data S2

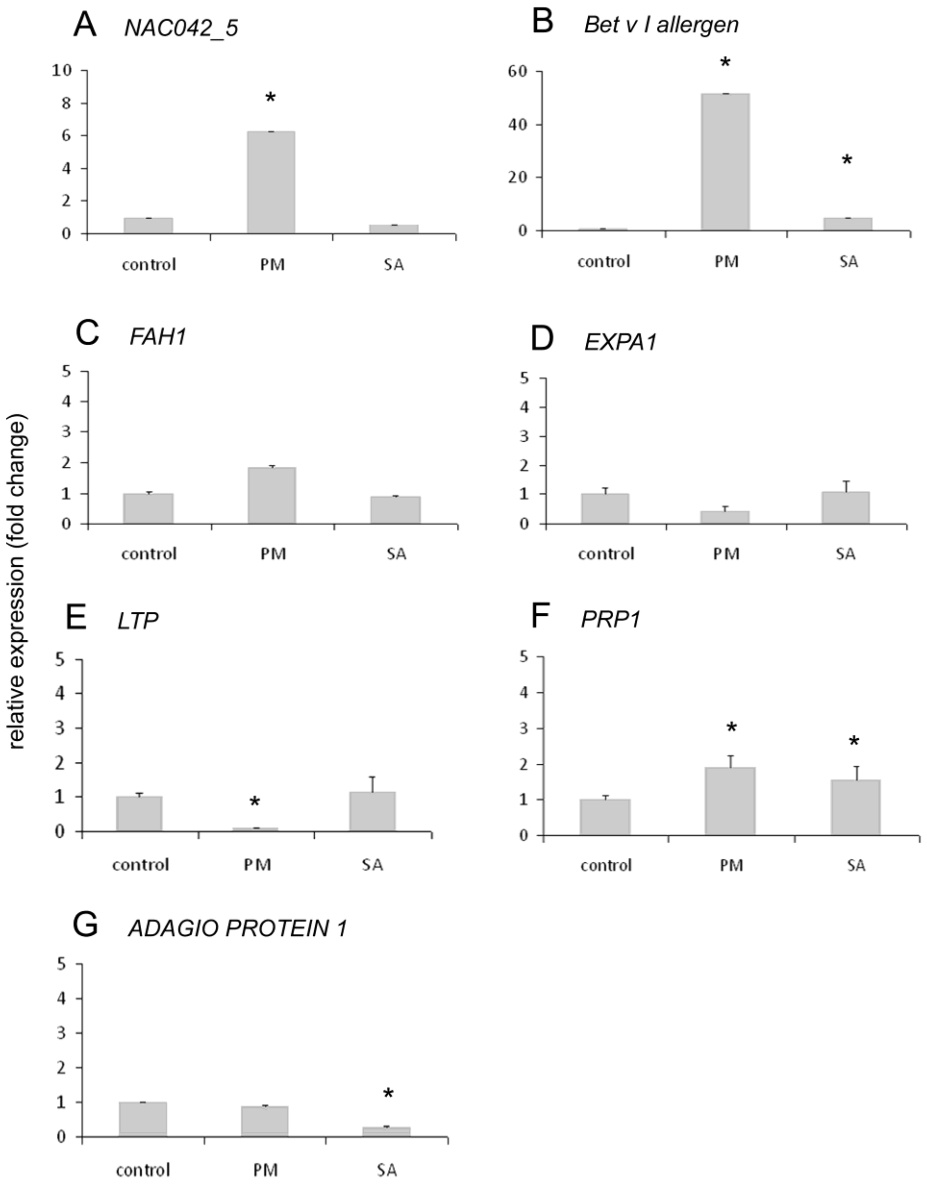

S2 Figure 1. Regulation of selected genes in PM-infected and MeSA-treated plants analyzed by qPCR. Expression is displayed as relative expression, asteriscs indicate significant difference to the control (T-test; p = 0.05), error bars indicate the standard deviation of three biological replicates. PM, powdery mildew responsive; SA, salicylic acid responsive.

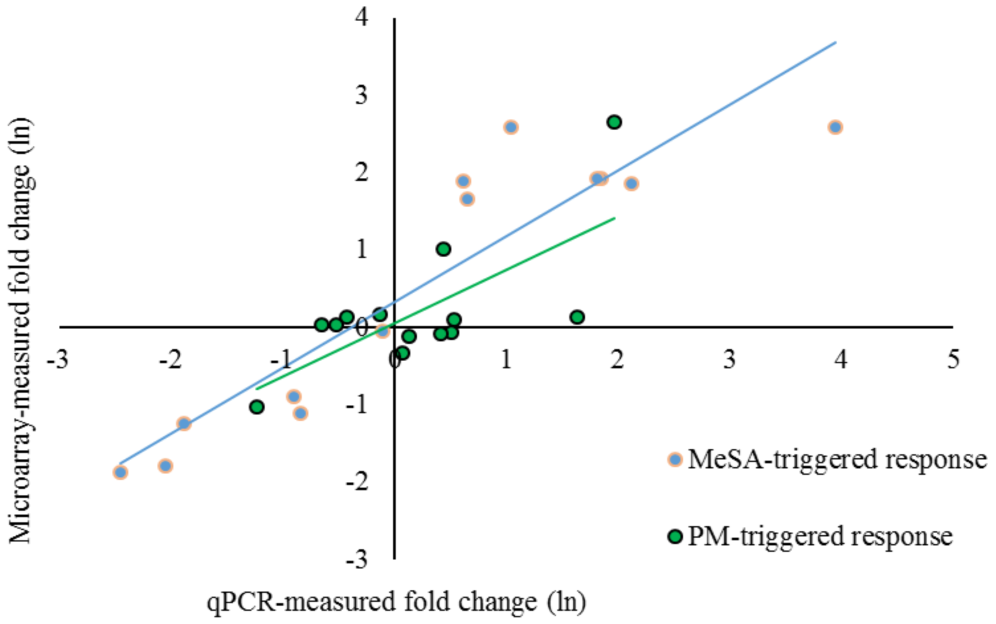

S2 Figure 2. Correlation between microarray and qPCR expression data (correlation coefficient $r^2 = 0.762$). qPCR validation was performed in samples from experiments repeated independently from microarray study.

S2 Table 1. Primer sequences used in the qPCR experiment.

Grape gene ID	Affymetrix Gene ID	Gene name	Primer F	Primer R
18864001	1613141_at	NAC042_5	TGGTTGTCGGCCAAATGAG	CAGTCATAAACATGAGGTGG
33089001	1618568_s_at	Bet v I allergen	CATCTATCAGTCTCAATAAGTTTGG	AAGGTAATACAATAATGACACTTCATG
19415001	1620245_at	FAH1	TGTATCATTGCCAACTGAGTTC	ATGACCTACCAAAGTAAGAG
20991001	1619082_at	EXPA1	TGTCCATGCTGTTTCCATC	GCTGGTGACTACTCTGAAG
32674001	1606656_at	LTP	CTTATTAGTGCGTGTGGG	CCGTTAACAACAGACAACAG
38581001	1611058_at	PRP1	TGGCTACCTACGCCCAGAAC	CGGTGCCTGTCAATGAAG
TC89970	1614760_at	ADAGIO PROTEIN 1	CTTCTGACGTGTTCCACTG	CACAACCACATCCCACG
TC60835 TC2116 TC9040 TC13894 TC25236 TC45156		ACTIN	CCCCACCTCAACACATCTCC	TCCATTGTCCACAGGAAGTGC
